# Intelligent Logistics System Design and Supply Chain Management under Edge Computing and Internet of Things

**DOI:** 10.1155/2022/1823762

**Published:** 2022-09-16

**Authors:** Tianxia Wang, Hong Chen, Rui Dai, Delong Zhu

**Affiliations:** ^1^School of Economics and Trade Management, Yibin Vocational and Technical College, Yibin 644003, China; ^2^School of Management, Guangzhou Xinhua University, Dongguan 523133, China; ^3^College of Finance and Statistics, Hunan University, Changsha, China; ^4^School of Economics and Management, Hubei University of Automotive Technology, Shiyan 442002, China

## Abstract

In order to carry out practical innovation of the intelligent logistics system and promote the practicality of the intelligent logistics system and supply chain management process, this study aims to optimize the design of the intelligent logistics system and supply chain management under edge computing (EC) and the Internet of Things (IoT). The flower pollination algorithm performs the positioning function in the intelligent logistics system and supply chain management. Based on the research on the design of the intelligent logistics system and supply chain management under the EC and IoT, this thesis analyzes the positioning of intelligent logistics systems and supply chain management through the flower pollination algorithm. The eXtreme Gradient Boosting (XGBoost) model is used to predict user information in the system of supply chain management information. Finally, the operation of intelligent logistics and supply chain management systems, the prediction model of supply chain management under XGBoost, and the change of supply chain management and material flow are analyzed. The results show that with the increase in the number of iterations, the optimized algorithm improves the comparison distance error by 53.57%, which has high accuracy and can meet the requirements of positioning and tracking of the intelligent logistics system and logistics status query in supply chain management. The waiting time of the intelligent logistics system is shorter than that of the supply chain management system, and the average waiting time of the system increases by 121.252 ms. The XGBoost model can well predict user information under supply chain management. After discussing the changes of the intelligent logistics system from 2018 to 2020, it is found that the operation efficiency of the supply management system is higher with the increase of the system operation days. The intelligent logistics system has a significant impact on the development of the logistics industry. This research gives a reference for establishing the intelligent logistics system and supply chain management system.

## 1. Introduction

Logistics is the basis of the development of the Internet of Things (IoT). As an economic activity, it appears with the emergence of commodities and develops with the development of commodity production [1]. The development of the IoT is dependent on the support of logistics. It can be said that logistics is one of the earliest industries in the IoT. Many logistics systems involve some advanced technologies, such as informatization, digitization, networking, integration, intelligence, flexibility, agility, visualization, and automation. Originally, the IoT is called the sensor network and used in the logistics industry. For example, radio frequency identification (RFID) is one of the most basic applications of the IoT [2–4]. In traditional storage, manual scanning is needed to obtain data and the efficiency is low. The storage location is not defined clearly, which causes the phenomenon of the random stack of goods. After the IoT is applied to the traditional warehousing management system, an intelligent warehousing management system is obtained. It can improve the efficiency of scoring and delivering goods, expand the storage capacity, reduce the labor intensity and cost, monitor the goods delivery process, and complete the data query [5–8]. Through the systematic management of logistics vehicles, the freight cars and goods can be tracked and monitored in real time, and the temperature and humidity of goods can also be detected. In the process of transporting goods, the vehicle speed, fuel consumption, tire temperature, and other driving behaviors are monitored in real time. In this way, the transportation efficiency is improved, and the transportation cost and loss are reduced [9–11].

With the combination of Internet technology and the logistics industry, the demand for intelligent logistics markets is increasing. China's modern logistics and supply chain management are still in their infancy. The suppliers only provide goods and have no value-added services [12], and the income of logistic companies and outsourcing service providers mainly comes from basic services (transportation management and warehouse management) and value-added services (supply chain integration services, supply chain financial services, and supply chain platform construction) [13, 14]. Google Cloud IoT is a platform designed for intelligent services, likefully hosted integrated services, which can be used to easily and safely connect to a large number of devices distributed around the world, manage and extract the data on these devices, visualize these data in real time, improve business agility and speed up decision-making, and make business changes [15, 16]. Edge computing (EC) can transform the pattern of the manufacturing industry from the traditional centralized control mode to the mode of real-time collection and processing of network edge data [17]. Since 2017, Google has announced two related new products, namely hardware Chip TPU and software Cloud IoT Edge, which helps promote the development of edge networking devices. Google said, “cloud IoT edge can extend the powerful data processing and machine learning capabilities of Google cloud to billions of edge devices, such as robot arms, wind turbines, and oil rigs, so that they can operate the data from their sensors in real time and predict the results locally [18–20].” The standardization of EC gradually attracts the attention of major organizations and relevant working groups have been established to do research on it [21]. Regarding the importance of studying the intelligent logistics system, Çakmak et al. pointed out in the optimization study of the express logistics system that the current logistics system played a great role in improving the efficiency of transportation logistics symbolized by express delivery [22]. Yan and Li illustrated the application of Radio Frequency Identification (RFID) technology in logistics information systems, and demonstrated the application of technology in logistics information systems [23]. Da Silva et al. proposed that logistics system and supply chain management interact and relate each other, which is the technical basis of supply chain management process. It has transparency and security of information in rapid response and model construction [24]. Regarding the technological development of intelligent logistics system, Feng and Ye realized intelligent transportation and warehousing operation process by optimizing the algorithm architecture of logistics information system in intelligent logistics system [25]. Li et al. proposed a control method and system under an intelligent logistics system [26].

Based on the research on the design of the intelligent logistics system and supply chain management under the EC and IoT, this thesis analyzes the positioning of intelligent logistics systems and supply chain management through the flower pollination algorithm. The eXtreme Gradient Boosting (XGBoost) model is used to predict user information in the system of supply chain management information. Finally, the operation of intelligent logistics and supply chain management systems, the prediction model of supply chain management under XGBoost, and the change of supply chain management and material flow are analyzed. This thesis has certain reference significance for the establishment of intelligent logistics systems and supply chain management. The innovation of this study lies in using the flower pollination algorithm to carry out the positioning work in the process of intelligent logistics management and using the IoT and EC to design the intelligent logistics system and supply chain management process.

This research is divided into four sections.  Section 1 describes the development of the modern intelligent logistics system, explains the research of logistics in China and foreign countries, and explains the research method and research frame.  Section 2 is the theory and research method. Firstly, the edge computing theory is introduced. Secondly, the intelligent logistics system and supply chain management process are designed combined with the IoT and EC. Finally, the positioning in the process of intelligent logistics management is carried out through the flower pollination algorithm.  Section 3 mainly analyzes the operation of intelligent logistics system and supply chain management system, and the changes of supply chain management and logistics flow.  Section 4 mainly summarizes the results and puts forward the specific prospect of future research and development.

## 2. Method

### 2.1. Theory of Edge Computing

EC is a distributed architecture that moves the operations of applications, data, and services from the central node of the network to the edge [27]. It decomposes the large-scale services initially processed by the central node into smaller parts and distributes them to the edge nodes for processing. The edge node is closer to the user terminal device, which can speed up the data processing and transmission speed. Under this framework, the data can be analyzed closer to the data source, so the framework can process a large number of data. EC is a decentralized computing architecture. It can store the data where they are needed and make most of the calculations done in distributed nodes. “Edge” refers to any calculations and network resources between the data source and the cloud data center. For example, smart phones are the “edge” between individuals and the cloud, and gateways in smart homes are the “edge” between home devices and the cloud. The basic principle of EC is to calculate near the data source. It is an open platform that integrates the core capabilities of networks, calculations, storage, and application and provides edge intelligent services nearby the edge of the network near the object or data sources. The goal is to make any application or function closer to the execution device.

EC is a supplement and optimization of cloud computing. It is also an important supporting technology of the 5th generation mobile networks (5G) and IoT [28, 29]. Cloud computing is to calculate big data in the cloud computing center (Data Center), which can realize on-demand access. However, it cannot meet the needs of real-time data processing and the explosive growth of networking equipment in the era of 5G and IoT. As a supplement to cloud computing, EC can be used in local decision-making and other scenarios.  Table 1 shows the collaboration points between EC and cloud computing.

**Figure 1:**
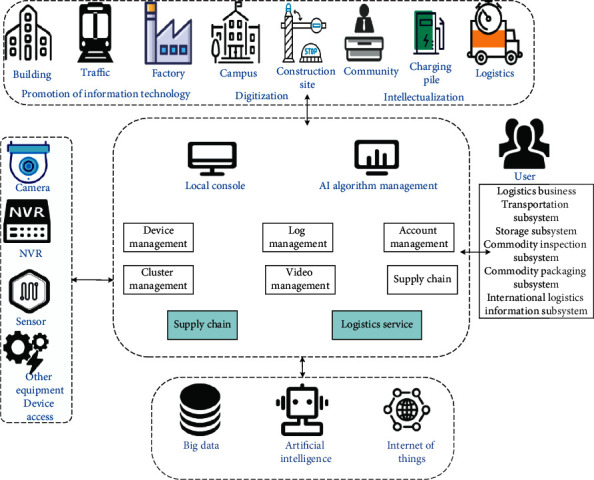
EC under IoT.

Table 1 shows the collaborative points of EC and cloud computing. The edge collects, stores, and preprocesses real-time data. After most redundant and unimportant data are excluded, only the cleaned data are sent to the cloud, reducing the network's pressure. It can store the data when the network signal is poor, and upload the stored data to the cloud until the network is restored. The cloud only needs to process and learn the massive data collected by each edge, and push the updated prediction model to the edge, which greatly relieves the workload of the cloud. Compared with cloud computing, EC is arranged nearby and can realize the unprecedented connectivity, centralization, and intelligence of IoT, which can meet the needs of agile connection, real-time business, data optimization, application intelligence, security, and privacy protection. It is an important support for realizing distributed autonomy and industrial control automation.  Figure 1 shows the process of EC under IoT.

Figure 1 shows the information integration solution provided by the EC under the IoT. It includes the servers, algorithms, applications, and equipment access capabilities needed to carry out edge services. The IoT edge computing provides cloud management and local management for all-in-one machines. The EC management platform under the IoT is a management console running in the hardware of an all-in-one machine. It can manage the offline all-in-one machine's network configuration, algorithm tasks, and terminal equipment. The one-click deployment function of the EC console can synchronize the resources and configurations between the cloud and the edge and view the deployment progress and trace the deployment log. The EC preloads the official application software in the all-in-one machine under the IoT and focuses on developing business logic without consuming energy, such as program startup, message flow, log query, and process maintenance. The EC under the IOT also supports the development of container applications or light applications to expand businesses. It also provides a multilanguage software development kit (SDK) to support common IoT devices and systems, including sensor devices, NVR (network video recorder), business systems, and other services connected to the cloud.

### 2.2. Intelligent Logistics System Based on IoT

The application of IoT in logistics system can improve automation, optimize transportation management process, improve the transmission of traffic information, and improve traffic efficiency. It can also reduce transport risk, improve resource utilization, reduce transport costs, and realize the automation, visualization, controllability, intelligence, and networking of logistics transportation. It enables all parties in transportation to share traffic information, and improves the integration ability of transportation enterprises and the ability to perceive and respond to market changes. It can timely and accurately provide customers with relevant product transportation information, and formulate optimal transportation plans for customers. It can also provide customers with the most satisfactory services and improve the overall logistics and transportation service level and service quality. The application of the IoT in supply chain management can enable supply chain system managers to accurately track and locate any item in any link of the supply chain, and achieve transparent supply chain management. Applying the IoT in supply chain management has achieved a high degree of integration of all aspects of the supply chain and improved the overall management efficiency of the supply chain.

**Figure 2:**
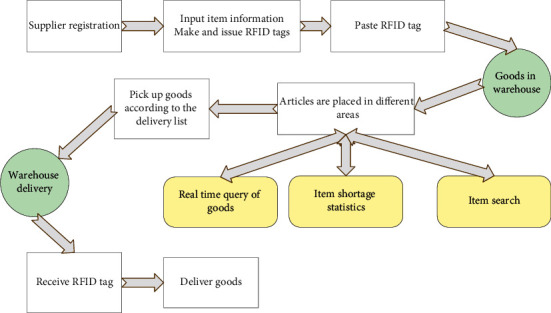
Design of the intelligent logistics system.

Intelligent logistics depends on the application of the Internet and IoT. It uses advanced technologies to collect, process, manage, circulate, and analyze data.  Figure 2 shows the design of the intelligent logistics system.

**Figure 3:**
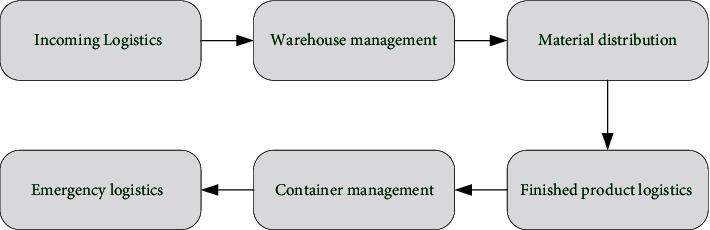
Overall framework of the intelligent logistics system.

Figure 2 shows the process of intelligently completing the packaging, transportation, distribution, loading, unloading, and warehousing. In this way, the flow status of goods is monitored in real time, so that the goods can be delivered to the demander efficiently and quickly, reducing the cost for the supplier. It can also significantly reduce the consumption of social and natural resources.  Figure 3 shows the overall framework of the intelligent logistics system.

In Figure 3, based on the business process of the intelligent logistics system, the overall framework of the informatization of the intelligent logistics system is clarified, and on this basis, the informatization function requirements of each link are proposed. It mainly includes inbound logistics, warehouse management, material distribution, finished product logistics, container management, emergency logistics, and other links. Meanwhile, it also clarifies the input, information drive, information collection, output, and other information of each process activity node.  Table 2 shows the functions of the intelligent logistics system.

**Figure 4:**
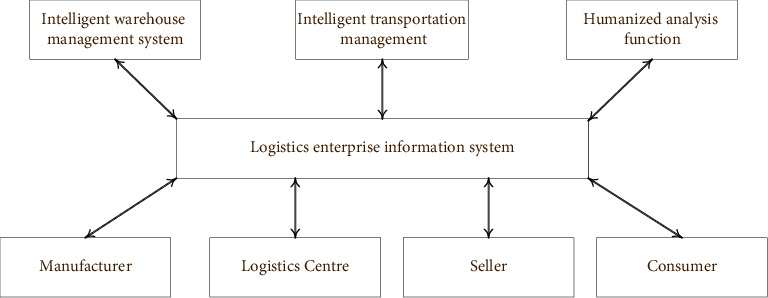
Framework of the intelligent logistics system.

According to the functions of the intelligent logistics system in Table 2, the design of the intelligent logistics system should meet the personalized and diversified needs of users.  Figure 4 shows the framework of the intelligent logistics system.

Figure 4 shows that the intelligent logistics system includes the advanced traffic information service system, vehicle control system, traffic management system, operating truck management system, and electronic toll collection system. ITS can realize logistics distribution management and centralized dynamic control of vehicles, including providing road traffic information, route guidance information to optimize the decision-making transportation scheme, accurate arrival time of vehicles through real-time tracking of vehicles, the warehouse inventory strategy, and distribution plan of the logistics center. This can realize the information sharing between each node of the logistics network and the headquarters and between network nodes, and improve the efficiency of the whole logistics transportation system. The IoT is a huge network that combines the global positioning system (GPS), infrared sensors, radio frequency identification (RFID) devices, laser scanners, and other devices with the Internet. Through this network, any object can be connected with the Internet for information exchange and communication, realizing intelligent identification, positioning, tracking, monitoring, and management.

### 2.3. Design of the Intelligent Logistics Supply Chain

**Figure 5:**
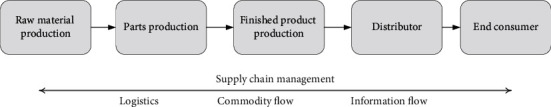
Process of logistics supply chain management.

Supply chain logistics management is the logistics management system centered on the core products or core businesses of the supply chain. The former focuses on the logistics management of the supply chain organized with the manufacturing, distribution, and raw material supply of core products as the system, such as the logistics management of automobile manufacturing, distribution, and raw material supply chain, which is the logistics management system centered on automobile products. The latter is the logistics management system of the supply chain organized with the core logistics business as the system, such as the logistics management of the third-party logistics, distribution, storage, or transportation supply chain. The logistics management of these two types of the supply chain has both similarities and differences. The core of supply chain management is the logistics management of the supply chain. Capital flows serve to create conditions to ensure the smooth progress of logistics.  Figure 5 shows the process of logistics supply chain management.

**Figure 6:**
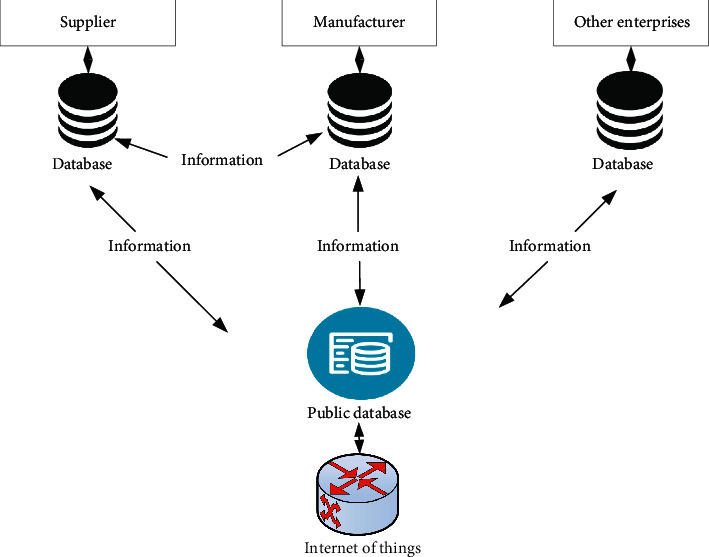
Information interaction mode of supply chain logistics management.

Figure 5 presents the logistics supply chain management business process, which is different from traditional logistics focusing on warehousing and distribution. The supply chain management covers the entire logistics link from raw material procurement to final distribution to end consumers and realizes the unification of logistics, information flow, and capital flow. The product manufacturing process provides a framework for how to develop new products and markets with suppliers. This process not only enables management to coordinate the flow of new products in the supply chain but also improves operations such as manufacturing, logistics, and marketing. Part production management process is a supply chain process, including all the necessary activities in the supply chain to obtain, implement, and manage the manufacturing flexibility, as well as the activities needed to move the product out of the factory. Distribution management processes based on customer value breakdown increase customer trust through customized product and service agreements. The supplier relationship management process formulates the healthy relationship between enterprises and suppliers. The process provides an important link in the supply chain among enterprises.  Figure 6 shows the information interaction mode of supply chain logistics management.

**Figure 7:**
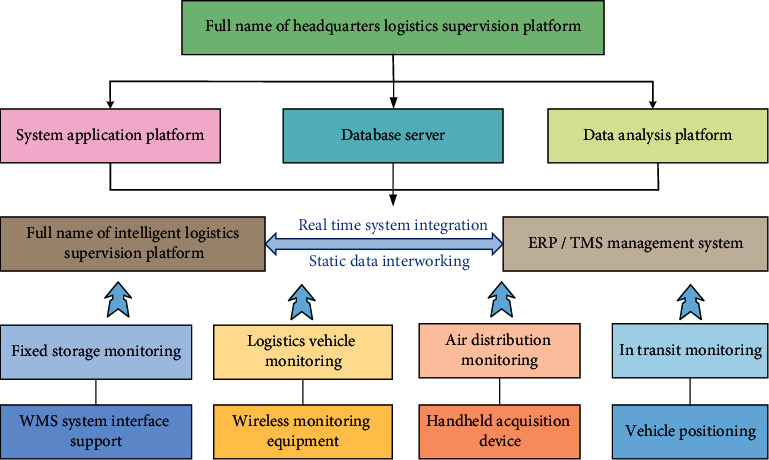
Operation process of the intelligent logistics management system in the supply chain.

Figure 6 shows that with the development of concurrent engineering, there are higher requirements for information management. The technology should support the collaborative work of multidisciplinary expert groups and realize the organic combination of interactive information, so that the correct information can be transmitted in real time. Therefore, in the actual process of information interaction based on SCLM, enterprises do not use a single mode for information interaction. They can select from the above three modes according to their needs and the confidentiality of the interactive information. On the basis of the first three interaction modes, the comprehensive information interaction mode integrates the interactive information to realize information integration and improve the efficiency of information interaction.  Figure 7 shows the operation process of the intelligent logistics management system in the supply chain.

Figure 7 shows that the intelligent logistics management system on the supply chain takes enterprise resource planning (ERP) and transportation management system (TMS) as the technical and theoretical support and traces the flow of goods on the whole supervision platform of the logistics head office. In addition, the platform reserves a data interface to facilitate docking with the customer system and has the functions of data analysis and report exports. The platform integrates human, material, information, and other resources, optimizes business processes, improves operation efficiency, connects the warehouse management system (WMS), and big data analysis platform of logistics enterprises, and realizes data and information sharing. The vehicle, equipment, and personnel information used in each link, as well as the temperature of the intelligent equipment in picking up, warehousing, transferring, and signing in, can also be displayed. The flower pollination algorithm is used to locate the process of intelligent logistics management. The relevant parameters are initialized, including flower population number *n* and conversion probability *p* [30, 31]. The pollen position is updated by the following equation:

(1) $$\begin{array}{c} X_{i}^{t+1}=X_{i}^{t}+L\left(g^{*}-X_{i}^{t}\right)\text{.} \end{array}$$

In equation (1), X_*i*_^*t*+1^ and *X*_*i*_^*t*^ are the solutions of *t *+* *1 and *t*, respectively, *g*^*∗*^ is the optimal global solution, and *L* is the step length. *L* is calculated by the following equation:

(2) $$\begin{array}{c} L∼\frac{\lambda \Gamma \left(\lambda \right)\sin\;\left(\pi \lambda /2\right)}{\pi s^{1+\lambda }},\left(s\gg s_{0}>0\right)\text{,} \end{array}$$

(3) $$\begin{array}{c} \Gamma \left(\lambda \right)=\overset{+\infty }{\underset{0}{\int }}x^{\lambda -1}e^{-x}dx\text{,}\left(\lambda >0\right)\text{.} \end{array}$$

In equation (2), Γ(*λ*) is a standard gamma function. When *λ*=3/2, the pollen is renewed and the boundary is crossed by

(4) $$\begin{array}{c} X_{i}^{t+1}=X_{i}^{t}+\in \left(X_{j}^{t}-X_{k}^{t}\right)\text{.} \end{array}$$

In (4), ∈ is a random number between 0 and 1, X_*j*_^*t*^ and *X*_*k*_^*t*^ are the pollen of different flowers of the same plant. Conversion probability *p* is a constant, and the adjustment of P is made by

(5) $$\begin{array}{c} p=0.8+0.2rand\text{.} \end{array}$$

In (5), rand is a random number between 0 and 1, and step factor *γ* of *L* is calculated by

(6) $$\begin{array}{c} \gamma =\frac{N-t}{N}\text{.} \end{array}$$

In (6), *N* is the maximum times of iterations, and *t* is the current times of iterations. Global search is conducted by (7). The random number of rand is modified during the local search to improve the flower pollination algorithm. The specific changes are made using

(7) $$\begin{array}{c} X_{i}^{t+1}=X_{i}^{t}+\gamma L\left(g^{*}-X_{i}^{t}\right)\text{,} \end{array}$$

(8) $$\begin{array}{c} \varepsilon_{t+1}=\left\{\begin{array}{cc} \varepsilon_{t}+ran\;d*\varepsilon_{u}\text{,} & rand<0.1\text{,}\\ \varepsilon_{t}\text{,} & rand>0.1\text{.} \end{array}\right. \end{array}$$

### 2.4. Prediction Model of Logistics Supply Chain Based on XGBoost

More and more enterprises use data analysis to deal with supply chain interruption and strengthen supply chain management. For example, the global COVID-19 pandemic highlights the strategic importance of an integrated supply chain as a response to disruption. The supply chain must be resilient and able to withstand shocks and accidents. Therefore, in the design of an intelligent logistics system, it is necessary to predict the important supply chain demand.  Table 3 is a machine learning algorithm for predicting the supply chain.

According to the XGBoost model in Figure 3, the prediction model in supply chain management is built. XGBoost is obtained based on the improvement of gradient boosting decision tree (GBDT). The XGBoost is also an additive model. If the given dataset has *n* samples and *m* features, the dataset can be expressed as *D*={(*x*_*i*_, *y*_*i*_)}. The predicted values of *K* functions are added to fit the model. (9) is the specific predicted value:

(9) $$\begin{array}{c} \hat{y}=\overset{K}{\underset{k=1}{\sum }}f_{k}\left(x_{i}\right)\text{,}f_{k}\in F\text{.} \end{array}$$

In (9), *F*={*f*(*x*)=*w*_*q*(*x*)_}(*q* : *R*^*m*^⟶*T*, *w* ∈ *R*^*T*^) is the function space constructed by all regression tree models. In terms of specific parameters, *q*(*x*) is a function that maps the eigenvector *x* to the leaf node index of the decision tree. *T* is the number of leaf nodes corresponding to a decision tree. *w* is the weight corresponding to the leaf node, so each tree *f*_*k*_ corresponds to a tree structure feature vector *q* and the weight vector *w* corresponding to the leaf node.

First, the regularization term is added to the objective function. The objective function equations in the algorithm are

(10) $$\begin{array}{c} L\left(\varphi \right)=\underset{i}{\sum }l\left(\hat{y},y_{i}\right)+\underset{i}{\sum }\Omega \left(f_{k}\right)\text{,} \end{array}$$

(11) $$\begin{array}{c} \Omega \left(f\right)=\gamma T+0.5\lambda w^{2}\text{.} \end{array}$$

*w*=(*w*_1_, *w*_2_, ⋯, *w*_*k*_). $l\left(\hat{y},y_{i}\right)$ is the difference between the predicted value and the real value. Ω is a regularization term, which uses the L2 norm of the number of leaf nodes and the corresponding value vector of leaf nodes to control the complexity of the trained tree model, and *λ* is the corresponding coefficient. The system inventory, diversion route, user, product, and user demand of the supply chain management's logistics system are used as input variables. (12) is the construction of characteristic variables:

(12) $$\begin{array}{c} v\left(t\right)=\frac{\overset{n}{\underset{j=1}{\sum }}m_{t-1}}{n}\text{.} \end{array}$$

In (12), *v*(*t*) is the created second manual input variable, *n* is the statistics of the number of times the user purchases a certain product, and *j* is the same user purchased such products for the *j* time. *m*_*t*−1_ refers to the last purchase of such products by the user. (13) is the use frequency of characteristic variables:

(13) $$\begin{array}{c} w\left(t\right)=\overset{p}{\underset{o=1}{\sum }}ir\text{.} \end{array}$$

In (13), *w*(*t*) is the use frequency of characteristic variable user information in week *t*. *p* is the total usage. *i* is the usage of characteristic variable user information in week *t*. *r* is the usage of all user information. The initial value of the maximum depth of the decision tree in the model's use is 10. Besides, the mean square error is used as the evaluation standard of the model. Then, the number of decision trees in the random forest is 200. When splitting, the minimum sample initialization value of the splitting node is 2, and the minimum sample initialization number of the cotyledon node is 1. The minimum initialization value of the sum of the weights of all the cotyledon nodes is 0. Moreover, the feature quantity in the splitting is automatic splitting. The maximum characteristic number index reads

(14) $$\begin{array}{c} m=log_{2}N\text{.} \end{array}$$

*m* is the maximum number of features, and *N* is the total number of features of the input variable. The mean absolute error (MAE), root mean squared error (RMSE), and average absolute percentage error are used to evaluate the prediction performance of this model in logistics supply chain management. (15) displays the MAE:

(15) $$\begin{array}{c} MAE=\frac{1}{m}\overset{m}{\underset{i=1}{\sum }}\left|h\left(x_{i}\right)-y_{i}\right|\text{.} \end{array}$$

*h*(*x*_*i*_) is the predicted demand value of the model for the *x*_*i*_-th sample point. *y*_*i*_ is the real demand data of the *x*_*i*_-th sample point. *m* is the number of predicted samples. (16) displays the RMSE:

(16) $$\begin{array}{c} RMAE=\sqrt{\frac{1}{m}\overset{m}{\underset{i=1}{\sum }}{\left(\left|h\left(x_{i}\right)-y_{i}\right|\right)}^{2}.} \end{array}$$

(17) is Spearman's rank correlation coefficient.

(17) $$\begin{array}{c} r_{s}=\frac{\overset{m}{\underset{i=1}{\sum }}\left[\left(h\left(x_{i}\right)-\bar{y}\right)\left(h_{1}\left(x_{i}\right)-{\bar{y}}_{1}\right)\right]}{\sqrt{\overset{m}{\underset{i=1}{\sum }}{\left(h\left(x_{i}\right)-\bar{y}\right)}^{2}}\sqrt{\overset{m}{\underset{i=1}{\sum }}{\left(h_{1}\left(x_{i}\right)-{\bar{y}}_{1}\right)}^{2}}}\text{.} \end{array}$$

In (17), −1 ≤ *r*_*s*_ ≤ 1. The larger the *r*_*s*_ is, the greater the correlation between the two feature information sequences IS. When *h*(*x*_*i*_) and *h*_1_(*x*_*i*_) are the values of the two feature information sequences at the *x*_*i*_-th sample point, respectively, $\bar{y}$ and ${\bar{y}}_{1}$ are the average values of the two feature information sequences.

## 3. Results and Discussion

### 3.1. Positioning and Tracking of the Intelligent Logistics System and the Logistics Status in Supply Chain

**Figure 8:**
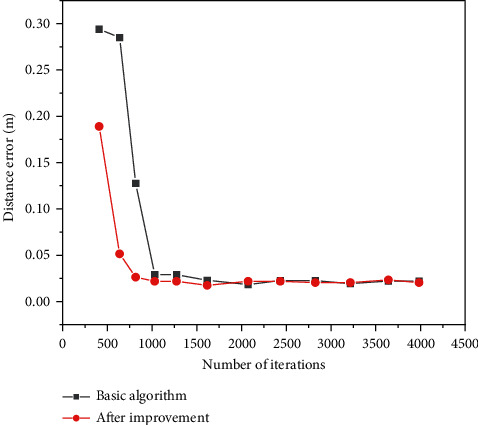
Distance errors of the flower pollination algorithms.

The intelligent logistics state is perceived according to the flower pollination algorithm. The distance errors of the products in the logistics system are compared before and after the optimization of the algorithm. The comparison results of the distance errors of the flower pollination algorithms are shown in Figure 8.

Figure 8 shows that when the number of iterations is 500, the distance error of the traditional flower pollination algorithm is 0.28 M, and that of the optimized algorithm is 0.13 M. This shows that the more the times of iterations are, the more stable the distance errors will be before and after optimization. With the increase of iteration times before and after optimization, the optimized algorithm has high accuracy in the distance error, which realizes the positioning and tracking of the intelligent logistics system and the logistics state query in the supply chain management.

### 3.2. Analysis of a Model of Supply Chain Management Prediction and Operation Efficiency in the Supply Chain Management System

**Figure 9:**
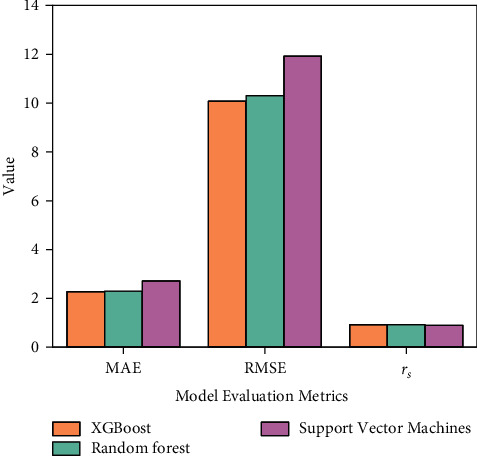
Error of the prediction model in supply chain management.

XGBoost is compared with random forest and support vector machine algorithms.  Figure 9 shows the error of the prediction model in supply chain management.

**Figure 10:**
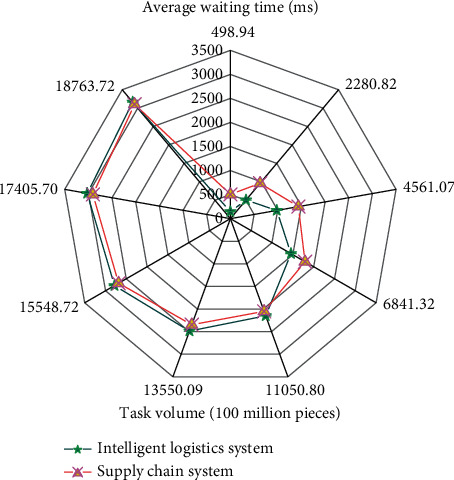
Average waiting time of the intelligent logistics system and supply chain management system in operation.

Figure 9 shows that there is little difference between the MAE value of XGBoost and that of random forest, indicating that the average fitting ability of XGBoost and random forest model in the prediction model of supply chain management is similar. The MAE value of the support vector machine model is the largest, indicating that the support vector machine model is not suitable for evaluating the prediction model. The RMSE of XGBoost is the smallest, which indicates that the predicted value of XGBoost *t* is the closest to the real value in the supply chain prediction model, and the numerical prediction ability of XGBoost is higher than that of the other two models. In addition, the fitting ability of the three models is good. The intelligent logistics monitoring system analyzes the data uploaded by each terminal when storing data in the cloud. The actual route and node time of goods transportation are displayed, so that the transportation status of goods is known. If the goods transportation route is set in advance, the alarm can be triggered when the goods deviate from the set route. The average waiting time of the intelligent logistics system and supply chain management system in operation is analyzed, as shown in Figure 10.

Figure 10 shows that the average waiting time of the intelligent logistics system in operation is 1855.379 milliseconds, and that of the supply chain system is 1976.631. This shows that the larger the amount of the task is, the longer the waiting time for system operation will be. In different periods, the waiting time of intelligent logistics system is shorter than that of supply chain management system.

### 3.3. Operation Efficiency of the Supply Chain Management System

**Figure 11:**
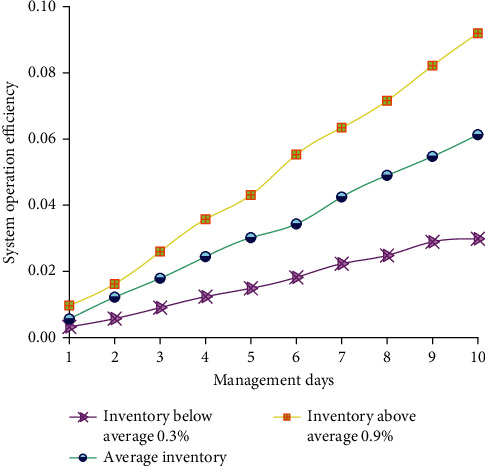
Operation efficiency of the supply chain management system.

The operation efficiency of the intelligent system and the supply chain management within 10 days is analyzed. After an in-depth analysis of the product transportation, route, and quantities, an exemplary management system is established based on the supply chain to realize the planning and management of the intelligent logistics supply chain.  Figure 11 shows the relationship between the supply chain management system and its inventory.

Figure 11 shows that the operation efficiency of the supply chain management system is compared and analyzed when its logistics inventory is at the average, 0.3% less than the average, and 0.9% less than the average inventory. It is found that the operation efficiency of the supply chain management system with an average inventory is lower than that of 0.3% less than the average inventory and higher than that of 0.9% less than the average inventory. With the increase of system operation days, the operation efficiency of the supply management system is more elevated.

### 3.4. Material Flow Changes of the Intelligent Logistics System

**Figure 12:**
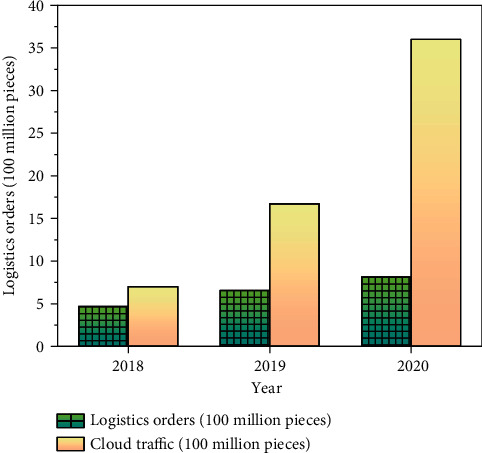
Changes of the material flow of the intelligent logistics system.

Based on the analysis of the changes of material flows of the intelligent logistics system from 2018 to 2020, intelligent cloud logistics iterate rapidly under AI, and the “intelligent revolution” changes the pattern of logistics markets. This shows that symbiosis and sharing are the developing trends, the service economy and experience economy are deepened, new ways of division and cooperation are widely used, and a win-win logistics ecosystem is established. It is estimated that by 2025, the revenue of the intelligent logistics market will exceed 1 trillion.  Figure 12 shows the changes in the material flow of the intelligent logistics system.

Figure 12 shows that intelligent cloud logistics develops rapidly from 2018 to 2020. Compared with Intelligent Cloud logistics, logistics orders are much fewer than traditional logistics systems. The difference in the revenue between the two is 233 million in 2018 and 2.788 billion in 2020. Intelligent logistics system has a significant impact on the development of the logistics industry.

## 4. Conclusion

The intelligent logistics system and the supply chain management system under the IoT are discussed based on EC, and the flower pollination algorithm is used to determine the positions in the system. When the times of iterations are 500, the distance error of the traditional algorithm is 0.28 M and that of the optimized algorithm is 0.13 M. The more the times of iterations are, the distance error tends to be stable before and after optimization. The average waiting time of the intelligent logistics system is 1855.379 milliseconds and that of the supply chain system is 1976.631 milliseconds. This shows that the larger the amount of the task is, the longer the waiting time for system operation is. XGBoost and random forest model have similar average fitting ability in the prediction model of supply chain management, while support vector machine model is not suitable for evaluating the prediction model. In this supply chain prediction model, the predicted value of XGBoost model is the closest to the real value, and the numerical prediction ability of XGBoost is higher than that of the other two models. The changes in the material flow of the intelligent logistics system from 2018 to 2020 show that with the increase of system operation days, the operation efficiency of the supply management system is higher. The intelligent logistics system has a great impact on the development of the logistics industry. This research gives a reference for establishing the intelligent logistics system and supply chain management system. However, with the rapid development of the IoT, the operating system in the logistics industry should be updated to adapt to the changes in the new era.

## Figures and Tables

**Table 1:** Collaborative points of EC and cloud computing.

Coordination points	EC	Cloud computing
Networks	Data aggregation	Data analysis
Businesses	Agent	Business arrangement
Application	Microapplication	Lifecycle management
Intelligence	Distributed reasoning	Centralized training

**Table 2:** Functions of the intelligent logistics system.

Realization elements	Content	Functions
Logistics function integration	Integrating logistics services and value-added services	Meeting the personalized needs of users
Real-time tracking of users	Understanding the changes in the user's needs	Improving service levels
Information integration	Other logistics information	Providing integrated and networked services
Financial integration	Financial supervision and management of logistics businesses	Achieving financial objectives

**Table 3:** Machine learning algorithm for forecasting supply chain.

Machine learning algorithm	Application	Algorithm advantages
Decision tree	Simple regression and classification	Low time complexity and high efficiency
XGBoost	Practical problems, data competition	Fast algorithm implementation speed and high operation processing speed
Random forest	Regression and classification tasks	Strong fitting ability and fast operation speed
Support vector machine	Data analysis and pattern recognition tasks	High fitting degree and high recognition rate

## Data Availability

The raw data supporting the conclusions of this article will be made available by the authors, without undue reservation.
